# Discriminating two bacteria via laser-induced breakdown spectroscopy and artificial neural network

**DOI:** 10.1186/s13568-023-01569-0

**Published:** 2023-06-20

**Authors:** Dina Arabi, Omnia Hamdy, Mahmoud S. M. Mohamed, Zienab Abdel-Salam, Mohamed Abdel-Harith

**Affiliations:** 1 Laser Applications in Metrology, Photochemistry and Agriculture Department, National Institute of Laser Enhanced Science, Cairo University, Giza, 12613 Egypt; 2 Engineering Applications of Lasers Department, National Institute of Laser Enhanced Science, Cairo University, Giza, 12613 Egypt; 3 Botany and Microbiology Department, Faculty of Science, Cairo University, Giza, Egypt

**Keywords:** Bacteria, LIBS, NELIBS, ANN, AgNPs

## Abstract

Rapid and successful clinical diagnosis and bacterial infection treatment depend on accurate identification and differentiation between different pathogenic bacterial species. A lot of efforts have been made to utilize modern techniques which avoid the laborious work and time-consuming of conventional methods to fulfill this task. Among such techniques, laser-induced breakdown spectroscopy (LIBS) can tell much about bacterial identity and functionality. In the present study, a sensitivity-improved version of LIBS, i.e. nano-enhanced LIBS (NELIBS), has been used to discriminate between two different bacteria (*Pseudomonas aeruginosa* and *Proteus mirabilis*) belonging to different taxonomic orders. Biogenic silver nanoparticles (AgNPs) are sprinkled onto the samples’ surface to have better discrimination capability of the technique. The obtained spectroscopic results of the NELIBS approach revealed superior differentiation between the two bacterial species compared to the results of the conventional LIBS. Identification of each bacterial species has been achieved in light of the presence of spectral lines of certain elements. On the other hand, the discrimination was successful by comparing the intensity of the spectral lines in the spectra of the two bacteria. In addition, an artificial neural network (ANN) model has been created to assess the variation between the two data sets, affecting the differentiation process. The results revealed that NELIBS provides higher sensitivity and more intense spectral lines with increased detectable elements. The ANN results showed that the accuracy rates are 88% and 92% for LIBS and NELIBS, respectively. In the present work, it has been demonstrated that NELIBS combined with ANN successfully differentiated between both bacteria rapidly with high precision compared to conventional microbiological discrimination techniques and with minimum sample preparation.

**Supplementary Information:**

The online version contains supplementary material available at 10.1186/s13568-023-01569-0.

## Introduction

As early as 460 B.C., Hippocrates reported a strong association between food consumed and human illness (Hutt and Hutt II 1984). Foodborne illness is usually due to the ingestion of a pathogen (food infection) or the toxin of a toxigenic microbe (food intoxication) with food. Nowadays, detecting, identifying, and quantifying disease-causing bacteria is very important to the consumer and the whole food industry. Outbreaks of food/water-borne illnesses frequently occur, especially with the emergence of drug-resistant bacterial pathogens. More than 200 different foodborne diseases have been identified (Mead et al. 1999). Therefore, there is a global need among food consumers, producers, processors, and researchers to develop fast, accurate, and easy-to-use bacterial detection methods.

Conventional detection of bacteria is mainly based on cultivation procedures using enrichment broths followed by isolating colonies on selective media, biochemical identification, and confirmation of pathogenicity. Accordingly, many efforts have been committed to identifying a reliable, fast detection and identification technique for microbiological samples like bacteria. Spectrochemical analytical methods are sensitive to the microorganisms’ membrane composition and could tell much about the bacterial identity and functionality. One of the first attempts to use spectroscopic analysis was made by Morel et al. in 2003 (Morel et al. 2003). He concluded that laser-induced breakdown spectroscopy (LIBS) could yield informative spectra to differentiate between six bacteria and two pollens in pellet form. LIBS is a spark sensor technology in which a few tens of short laser pulses (nanosecond, picosecond, or femtosecond) are focused onto a target (solid, liquid, or gas). In the case of solid targets, as a result of focusing such a tremendous amount of energy on a tiny volume, the material dielectricity breaks down, forming a transient plasma plume. Such a laser-induced plasma plume consists of ions and electrons at extremely high temperatures (6000–60,000 K). As the hot plasma plume cools down, it gets rid of the previously absorbed energy in the form of emitted light photons at different wavelengths. The spectroscopic analysis of such emitted light provides a spectrum composed of the characteristic spectral lines of the elements in the plasma plume and, consequently, in the analyzed target material. LIBS fundamentals and applications are detailed in numerous books and published review papers (Cremers and Radziemski 2013; Legnaioli et al. 2020; Noll 2012; Singh and Thakur 2007).

LIBS’s applications in diverse life science fields, e.g. health (Gaudiuso et al. 2019), veterinary (Abdel-Salam et al. 2017), biology (Abdel-Salam et al. 2019), food (Abdel-Salam et al. 2018; Hamdy et al. 2022), microbiology (Diedrich et al. 2007), etc. were highly appealing. Notably, the technique attracted many researchers to characterize and classify bacteria based on their elemental and/or molecular composition. The first attempt to use LIBS to identify bacteria based only on their atomic constituents was in 2003 (Morel et al. 2003). After that, LIBS has been utilized by many researchers extensively for bacterial pathogens identification and characterization (Lewis et al. 2011; Manzoor et al. 2014; Mohaidat et al. 2012). LIBS has advantages in this field of research compared to other conventional techniques; it needs little to no sample preparation, is fast, has the possibility of in situ and remote analysis, and is cost-effective. However, the technique is inferior in sensitivity compared to other well-established spectrochemical analytical ones. Therefore, experimental modifications have been proposed to overcome such drawbacks. To mention some, e.g. changing the ambient conditions, double-pulse LIBS (DP-LIBS) (Babushok et al. 2006), and combining LIBS + LIF (laser-induced fluorescence) (Telle et al. 2001).

One of the essential cons of LIBS is its low sensitivity compared to other well-established spectrochemical analytical techniques. In 2009, (Ohta et al. 2009) suggested a simple method to beat such deficiency of LIBS with minimum cost and complication in the equipment. They suggested utilizing surface plasmon resonance to improve LIBS performance in detection sensitivity. In 2013, De Giacomo et al. deposited metallic nanoparticles (NPs) on the sample’s surface and then focused the laser on it. Adopting this approach, they enhanced the LIBS spectral lines’ signal-to-noise ratio (SNR) of 1–2 orders of magnitude. Such an improved method has been referred to as Nanoparticle-Enhanced LIBS (NELIBS), successfully utilized in many other publications (Balaji et al. 2008; Chen and Fu et al. 2018; Koral et al. 2018; De Giacomo et al. 2013, 2014, 2016a,b; Koral et al. 2016; Poggialini et al. 2018). Biosynthesized NPs were advantageous because of their low cost compared to the commercially available or chemically made ones; therefore, many researchers utilized such green synthesized NPs (Abdel-Salam et al. 2018; Poggialini et al. 2018; Krishnaraj et al. 2010).

The laser-induced plasma plume in the case of NELIBS has a higher temperature and prolonged lifetime. Hence, the emitted spectral lines will be more intense, with effectively improved SNR leading to the possibility of detecting faint spectral lines that are barely detectable in the case of conventional LIBS.

Introducing chemometric tools has recently allowed better manipulation and analysis of spectral data in classifying, identifying, and detecting biological materials (Granato et al. 2018). For example, it was reported by Multari et al. that LIBS combined with the appropriate chemometric models was influential in differentiating between two types of bacteria (Multari et al. 2010). Likewise, Marcos-Martinez et al. reached over 95% discrimination accuracy using LIBS combined with neural networks (N.N.s) to identify *Pseudomonas aeruginosa*, *Escherichia coli*, and *Salmonella Typhimurium* (Marcos-Martinez et al. 2011).

The current study presents a detailed investigation of using NELIBS compared to LIBS for differentiation between two economically important bacteria belonging to two-unlike taxonomic ranks. In addition, an artificial neural network (ANN) analysis of the spectroscopic data was utilized to compare the sensitivity of the techniques to bacterial variation.

## Materials and methods

### Bacterial samples

This study used two Gram-negative bacterial strains belonging to the taxonomic class *Gammaproteobacteria*. The first bacterial sample was an authenticated strain obtained from the American Type Culture Collection (ATCC), *Pseudomonas aeruginosa* (ATCC 9027), which belongs to the order *Pseudomonadales*. The second bacterial sample, *Proteus mirabilis* (D31) from the order *Enterobacteriales* is a local isolate; it was isolated from chicken meat tenderloins bought from local markets in Al-Haram district, Giza governorate, Egypt. First, the isolate was identified using conventional microbiological methods. Then it was confirmed by Matrix-assisted laser desorption/ionization-time of flight mass spectrometry (MALDI-TOF/MS) with score values > (19) (using Bruker Biotyper 3.1 software) and molecular analysis of 16SrRNA gene sequence. The final sequence was submitted to the GenBank database under accession number OK178865. Furthermore, it was deposited in the Culture Collection Ain Shams University (CCASU) (http://www.wfcc.info/ccinfo/detail) under the number (CCASU-2023-43).

Both bacterial strains were stored and maintained at 4 °C on Luria–Bertani (L.B.; Conda SA, Madrid, Spain) agar slants and in glycerol (20%, v/v) at − 80 °C. In addition, bacteria were recovered on L.B. broth medium overnight at 37 °C and then subcultured on tryptone soya agar (TSA; Conda SA, Madrid, Spain) plates to obtain single colonies.

A single colony from each bacterial culture was suspended in 200 µL deionized sterile water, adjusted to 0.5 MacFarland. The 0.5 McFarland bacterial suspension is a standardized method to adjust bacterial cell density to ~ 1.5 × 10^8^ CFU/mL. It was controlled by measuring the optical density of the bacterial suspension to obtain O.D. = 0.09 and counting the bacteria by the CFU method for LIBS measurements. After that, 100 µL of the bacterial suspension was put onto a pure Si wafer’s polished surface as a one-cm diameter droplet to allow numerous laser shots for the same sample. Finally, samples were left to dry in an oven for 5 min at 50 ℃ to evaporate the water and form a dry layer of bacterial suspension attached to the solid surface for easier sample manipulation.

### Biogenic silver nanoparticles

The silver nanoparticles used in this study were biosynthesized using the extract of *Streptomyces catenulae* (Kamel et al. 2016). The biosynthesis of silver nanoparticles was performed as follows; *Streptomyces catenulae* strain 24 was incubated on starch casein agar medium (Thermo Fisher, USA) for five days at 28 ℃ pH 7.0. First, the actinobacterial culture was centrifugated, and an equal volume of the cell-free supernatant was mixed with 1% silver nitrate (v/v) in a dark glass bottle. Then the mixture was incubated at 30 ℃ for 24 h on an orbital shaker at dark conditions. After the biosynthesis, the silver NPs were centrifuged at 10,000 rpm for 15 min and washed thrice with deionized water. After separation, the formed nanoparticles were lyophilized, and the nanoparticles were weighed per 100 mL of deionized water. The nanoparticles (NPs) size ranged between 10 and 20 nm (see the TEM micrograph in Additional file 1: Fig. S1) with a mass concentration of 50 µg/L suspended in deionized filtered water. In the case of NELIBS, 100 µL of the NPs was deposited onto the surface of the dried-up bacterial film to cover its whole area. Then, samples were placed in an oven for 5 min at 50 ℃ to evaporate the water and form a coating layer of NPs onto the bacterial sample’s surface.

### LIBS instrumentation

**Fig. 1:**
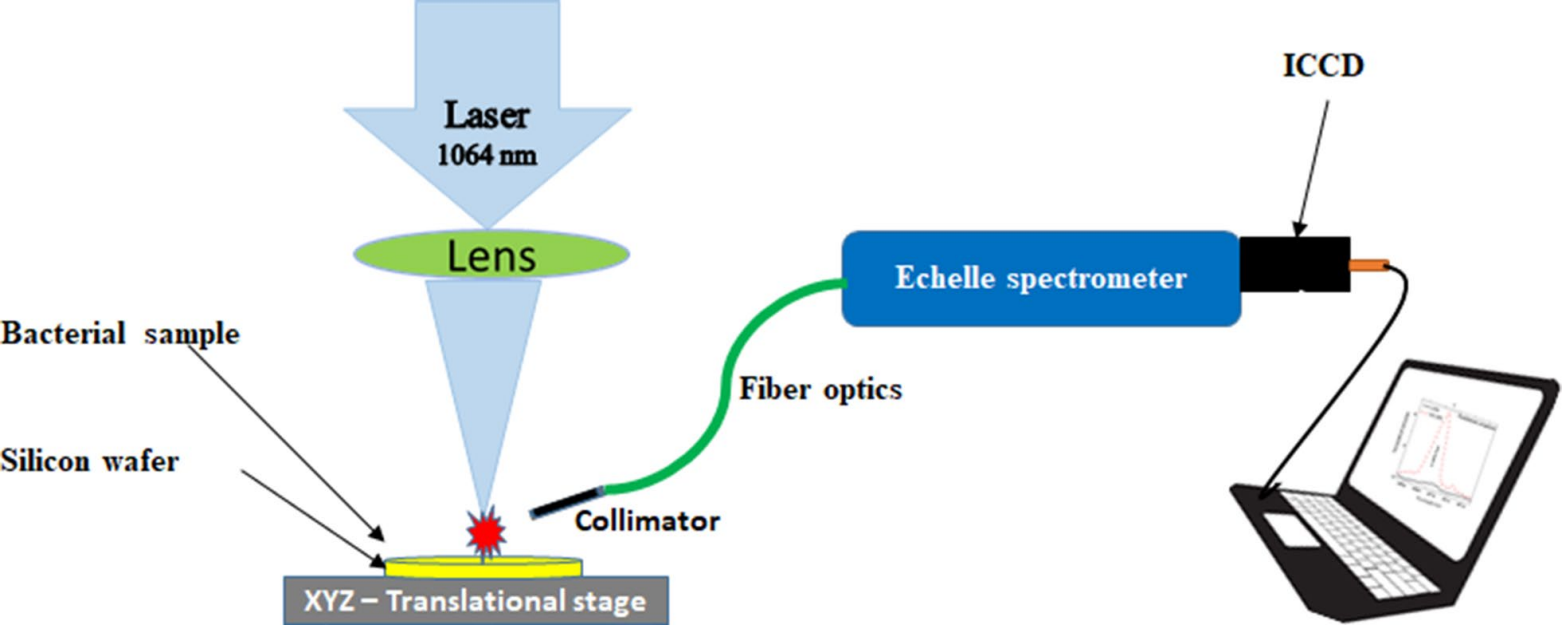
The schematic diagram of the experimental setup

Figure 1 depicts a schematic diagram for the experimental LIBS setup used in the present work. The LIBS measurements were conducted using a Q-switched Nd: YAG laser (Brio, Quantel, France). The laser beam was optimized at a pulse duration of 5 ns, 20-Hz repetition rate, a wavelength λ = 1064 nm, and pulse energy of 40 mJ monitored by a Joulemeter (SciTech, model AC5001, Boulder, CO, USA). A quartz planoconvex lens with a focal length of 5 cm was used to focus the laser beam onto the sample surface. The sample was fixed on an X–Y–Z micrometric translational stage that allowed movement along the sample area and controlled the focal distance to confirm breakdown onto the sample surface. The photons of the emitted plasma were collected by an optical collimator coupled to a silica fiber with a core diameter of 600 µm and fed to an echelle spectrometer (Mechelle 7500, Multichannel, Sweden). An ICCD camera (DiCAM PRO, PCO-Computer optics, Germany) was coupled to the spectrometer to detect the dispersed light.

### Data acquisition

Thirty LIBS spectra were collected from various spots on each sample. Each spectrum represents the accumulation of 3 laser shots taken at different positions (1 laser pulse per location). Spectra were collected at a delay time of 1500 ns after firing the laser pulse and a gate width of 3000 ns. Each data set was averaged to represent an individual spectrum for each sample. The spectral lines of interest were identified using LIBS +  + software (Corsi et al. 2001). The spectral lines were selected based on the signal-to-noise ratio (Calculated as the peak signal divided by the average of the surrounding background signal), the absence of overlapping, and the National Institute of Standards and Technology (NIST) spectral database confirmation of the spectral line wavelength. The best-resolved lines defined which elements could be used in the analysis. Spectral data manipulation and analysis were performed using OriginPro, Version 9 (Origin Lab Corporation, Northampton, MA, USA.) and Prism software, Version 5.01 (GraphPad Prism software, Inc.).

### The artificial neural network (ANN)

Artificial neural networks (ANN) are common computational methods that classify input groups according to target classes. The present study used the feed-forward network to classify the obtained LIBS spectra (the network’s input) with and without the nanoparticles based on Matlab R2018b software. A typical feed-forward network consists of a series of layers where the first layer connects to the network input, and each subsequent layer has a connection from the previous layer (Hamdy et al. 2022). At the same time, the final layer produces the network’s output. Using the scaled conjugate gradient (SCG) method, a common and efficient network training function named “trainscg” was selected to update the weight and bias values (Babani et al. 2016). Although the SCG algorithm is based on conjugate directions, it does not perform a line search at each iteration (which makes the system computationally expensive). Consequently, this approach was developed to eliminate the time-consuming queue search (Møller 1993).

**Fig. 2:**
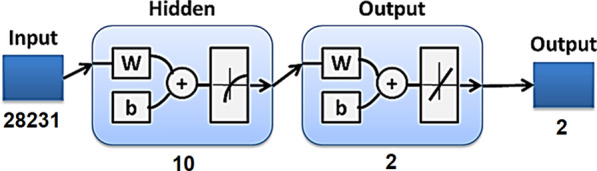
The Implemented Neural Network Model

The implemented ANN model was implemented using ten neurons in the hidden layer. The input data sets have been randomly divided into 70%, 15%, and 15% for training, validation, and testing; such a ratio is appropriate for the relatively small dimensional datasets (Rácz et al. 2021). The construction of the ANN model is shown in Fig. 2.

Based on trial and error, the number of neurons in the hidden layer of an ANN is selected. However, input nodes influence the number of neurons used in a particular scenario (Sheela and Deepa 2013; Stathakis 2009). In the current work, various neurons were considered in the ANN investigations to ensure optimum results. However, using the number of neurons in the hidden layer > 10 makes the ANN classification critical because the spectral inputs are too many to exclude the risk of overfitting. Therefore, ten neurons in the hidden layer were selected in our implementation. The entire set of spectra pixels (i.e., spectral lines) have been loaded into the proposed ANN model. There were 28231 input variables corresponding to wavelength ranges of 250 nm to 650 nm. 30 LIBS spectra for each type of bacteria with and without nanoparticles were used to construct the network.

## Results

### LIBS versus NELIBS in bacteria discrimination

**Fig. 3:**
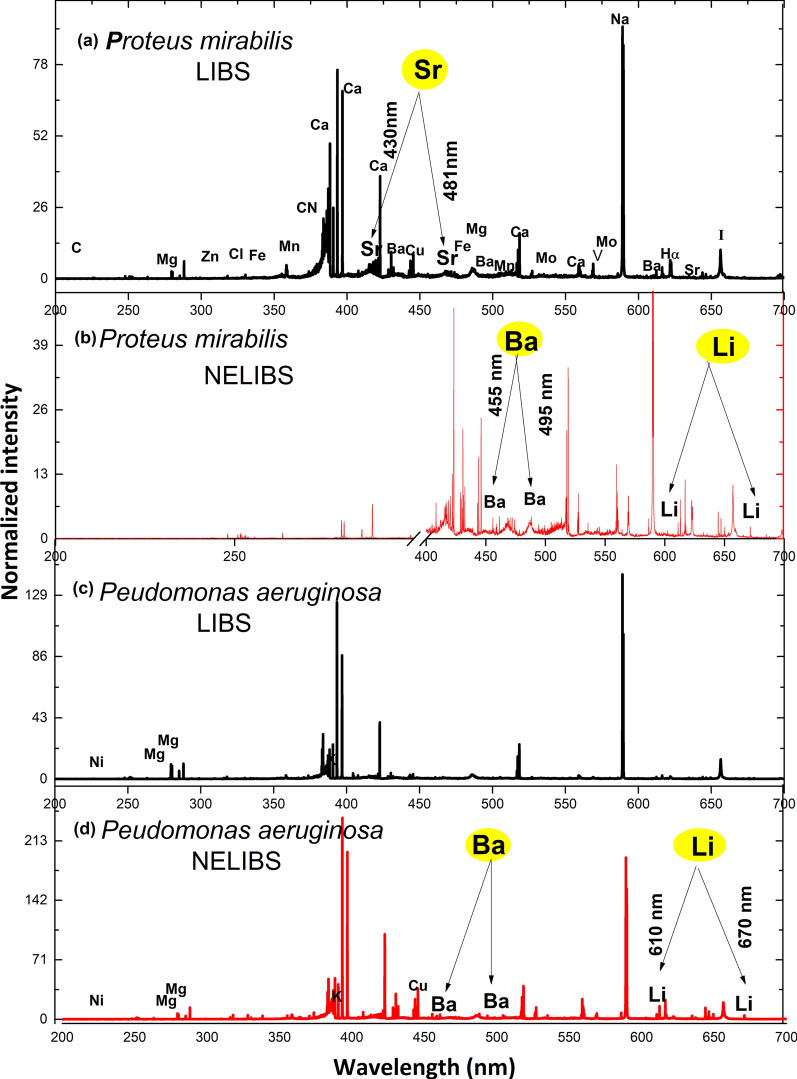
Average spectra for **a** *Proteus mirabils* LIBS, **b** *Proteus mirabilis* NELIBS, **c** *Pseudomonas aeruginosa* LIBS, **d** *Pseudomonas aeruginosa* NELIBS with the most significant element lines identified

**Fig. 4:**
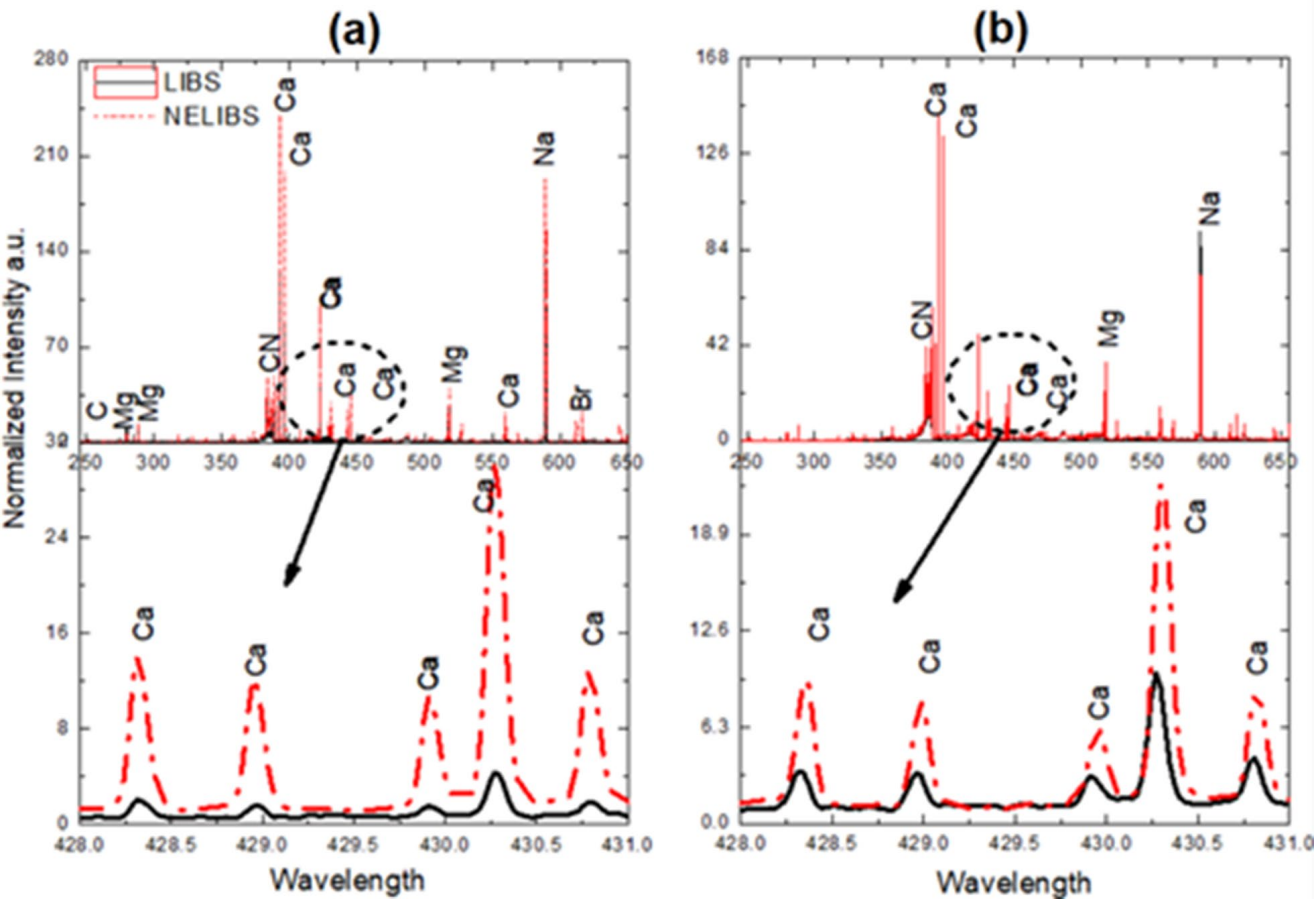
Upper: typical LIBS and NELIBS spectra of *Proteus mirabilis* (**a**) and *Pseudomonas aeruginosa* (**b**). Lower: zoomed section for each

LIBS and NELIBS spectral signatures of both *Pseudomonas aeruginosa* and *Proteus mirabilis* were obtained from 200 to 700 nm. As seen in Fig. 3. Numerous spectral lines show up in the sample spectra, e.g., Na, Mg, Mn, Fe, Mo, Sr, K, and Cl. Moreover, spectral lines of some trace elements appeared, though faint, such as Ni, Zn, Al, Cu, Ba, Li, and V. Besides, C.N. molecular bands show up in the sample spectra. The displayed spectra represent the averages of 30 LIBS and NELIBS spectra for both bacterial samples. Spectra were normalized to the intensity of the C 247.85 spectral line to minimize the effect of background noise. Figure 4 shows a pronounced signal intensity enhancement represented by five Ca spectral lines for the NELIBS spectra compared to LIBS that show up in both samples.

**Fig. 5:**
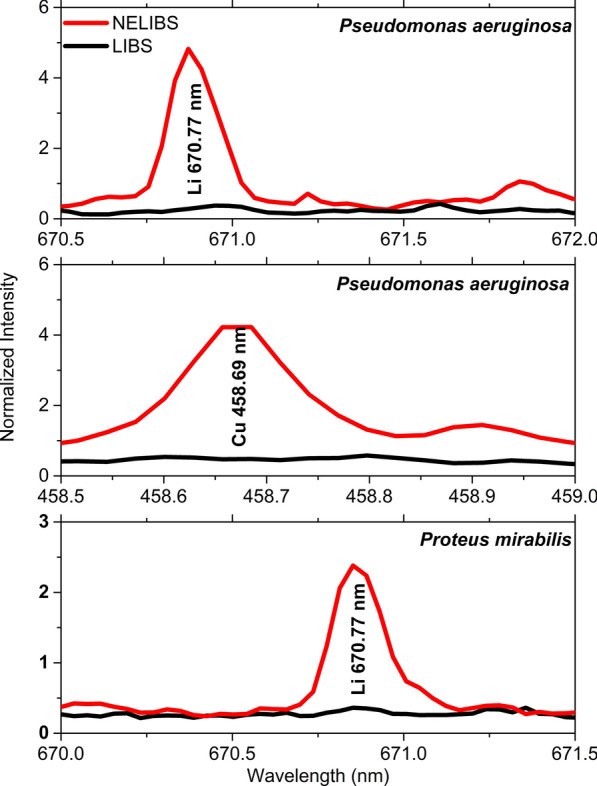
A comparison between LIBS and NELIBS of Li-670.7 nm, Cu 458.69 nm spectral lines for *Pseudomonas aeruginosa*, and Li-670.7 nm spectral line for *Proteus mirabilis*

Figure 5 shows some trace elements’ spectral lines that were detectable only by NELIBS. Namely, e.g. Li-670 nm for both *Pseudomonas aeruginosa* and *Proteus mirabilis* and Cu-458.69 nm for *Pseudomonas aeruginosa*.

**Fig. 6:**
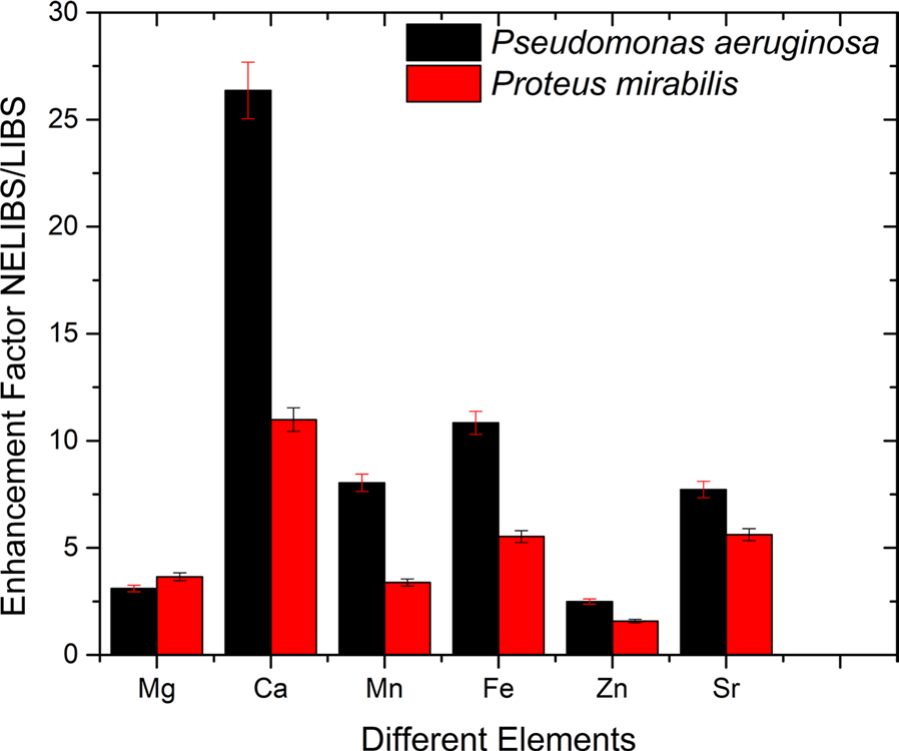
The intensity enhancement factor (NELIBS/LIBS) for selected spectral lines for both bacterial species. The error bars represent the standard deviation of the experimental data

The spectral lines intensity varies among the two bacterial species in the LIBS and NELIBS spectra. The average of the line’s intensity of six elements, Mg, Ca, Mn, Fe, Zn, and Sr, in 20 spectra for both LIBS and NELIBS, have been selected to demonstrate the spectral lines enhancement factor (ratio of NELIBS/LIBS for the intensity of the spectral lines). The bar graph in Fig. 6 shows that the enhancement factor differs for the chosen elements and is systematically higher for *Pseudomonas aeruginosa* than *Proteus mirabilis*. The matrix effect, i.e. the difference in the elemental composition proportions of each bacterial species, may affect such variation in the spectral line’s enhancement factor between the two bacterial species.

### Presence and absence of some elements’ spectral lines in NELIBS spectra

**Fig. 7:**
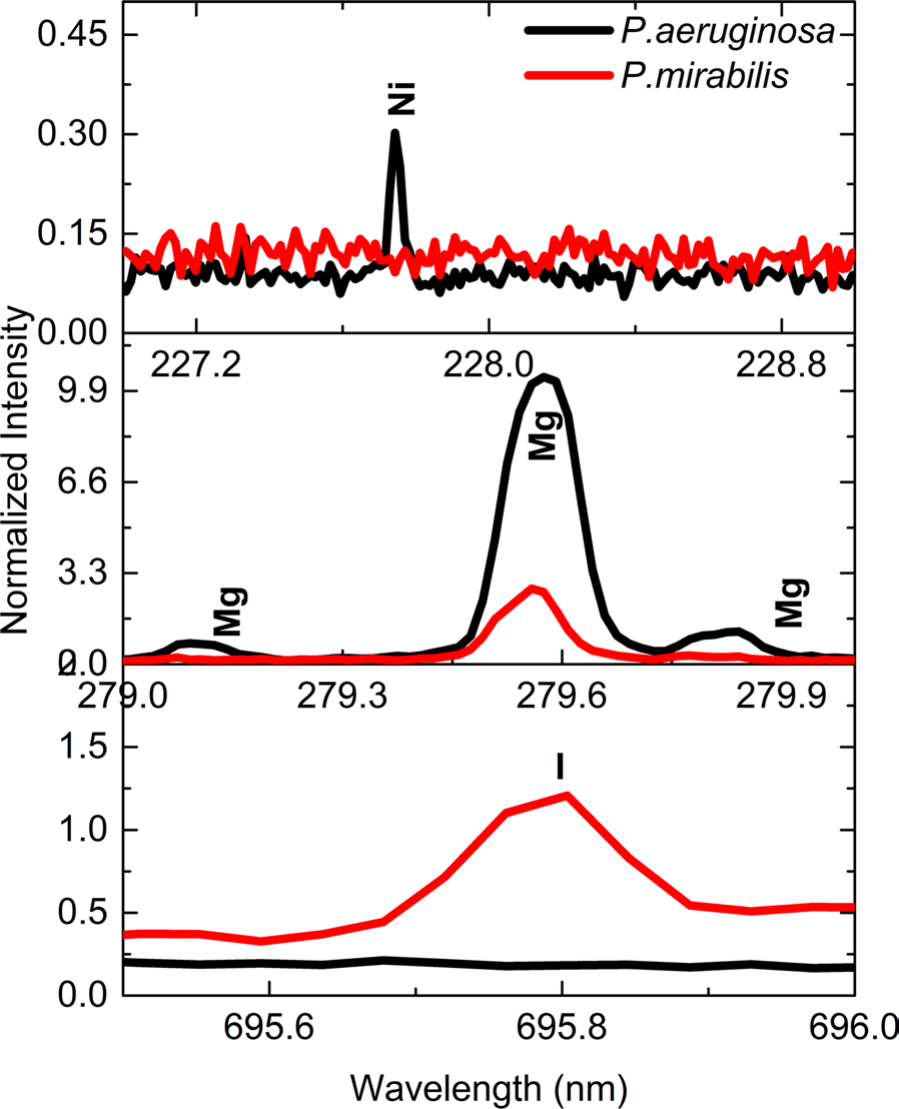
The iodine, magnesium, and nickel spectral lines in the NELIBS spectra of the *Pseudomonas aeruginosa* and *Proteus mirabilis*

The superiority of NELIBS compared to LIBS has been demonstrated for the spectral discrimination between the two bacterial species under study. Adopting NELIBS, the spectral lines of some elements, e.g. I, Ni, and Mg, could not or are barely detectable in the spectrum of one of the two species. On the other hand, these lines show up clearly in the spectrum of the other species, see Fig. 7.

### Artificial neural network (ANN) results

The constructed ANN was implemented twice, the first for raw samples (without nanoparticles) and the other after adding nanoparticles to the samples under examination. The accuracy rate of the constructed ANN models is presented in Table 1. As depicted in the table, without using NPs, the abstained accuracy rates were 94%, 79%, 65%, and 88% for training, validation, testing, and all data sets, respectively. While using the NPs, the accuracy rate increased to 92% for all data sets. According to the obtained confusion matrix, other evaluation indicators, including precision, recall, and F1-Score, are also provided in the table.

**Table 1:** ANN Implantations results with and without adding nanoparticles to the examined samples

Samples	Number of neurons	Accuracy rate				Precision	Recall	F1 score
		Training (%)	Validation (%)	Test (%)	All (%)			
Without nanoparticles	10	94	79	65	88	0.88	0.90	0.88
With nanoparticles	10	95	90	80	92	0.92	0.93	0.92

**Fig. 8:**
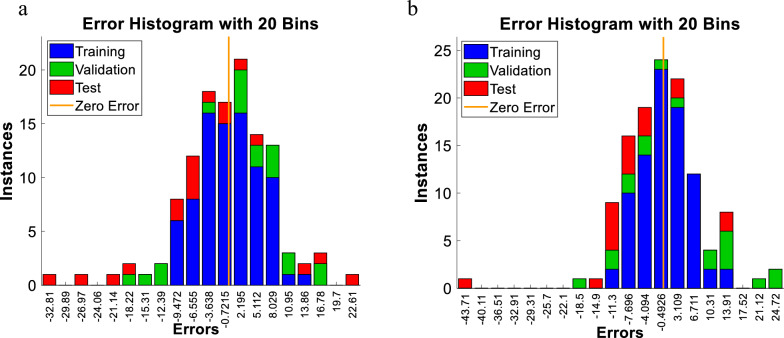
Error histogram plot of training, validation, and testing for one of the ANN executions **a**) using LIBS, **b** using NELIBS

A sample of the error histogram obtained from the implemented ANN models is presented in Fig. 8.

**Fig. 9:**
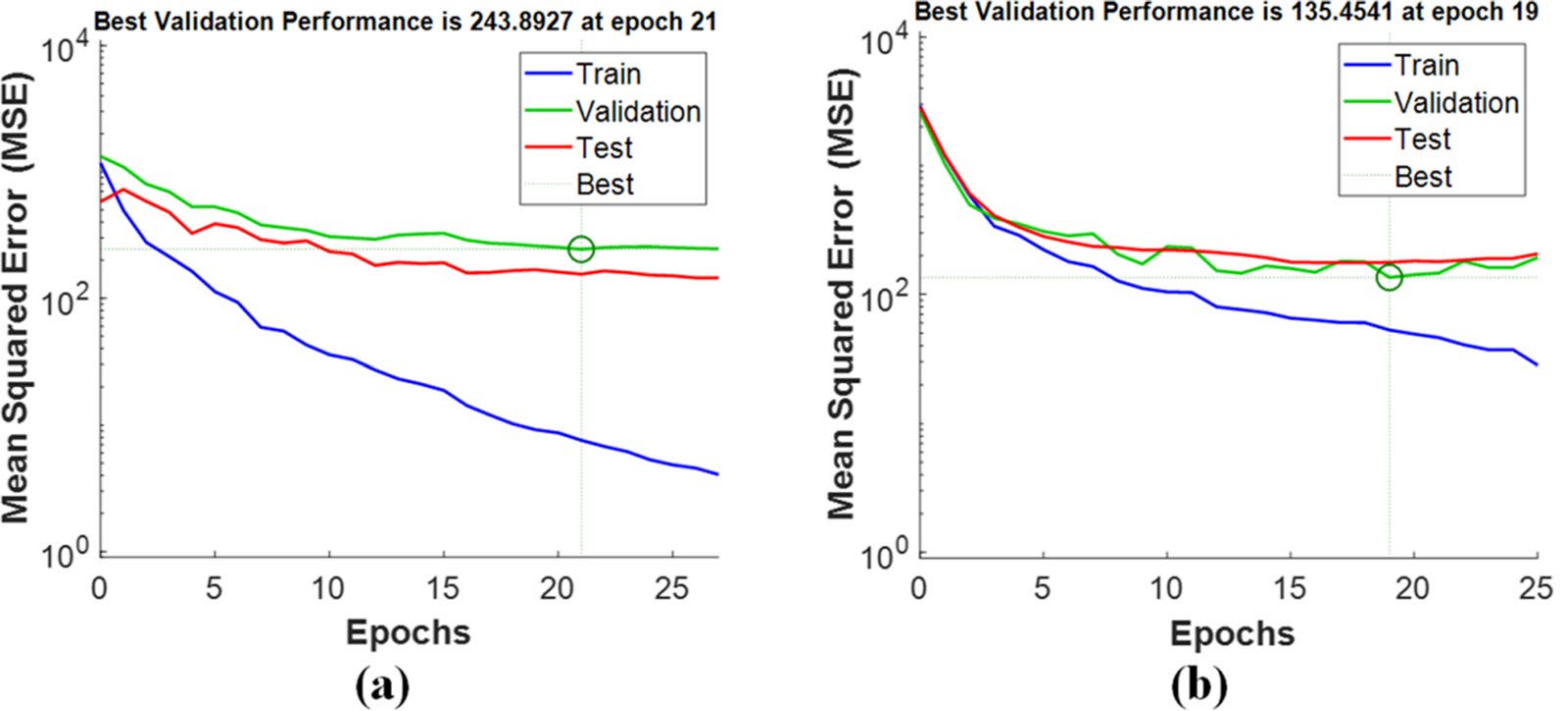
Mean Squared Error (MSE) graph of the trained ANN model (i.e. performance plot with best mean square error) **a** using LIBS, **b** using NELIBS

The error presents the difference between the desired and the real output produced by the network. The “Instances” in the figure refer to the number of training, validation, and test sets samples. It is clear from the figure that the fitting data errors are distributed near the zero-error region when using nanoparticles (Fig. 8b), verifying better performance upon adding NPs to the samples. Networks are run several times to obtain the best performance. The performance plot with minimum mean square errors (MSE) is presented in Fig. 9.

**Fig. 10:**
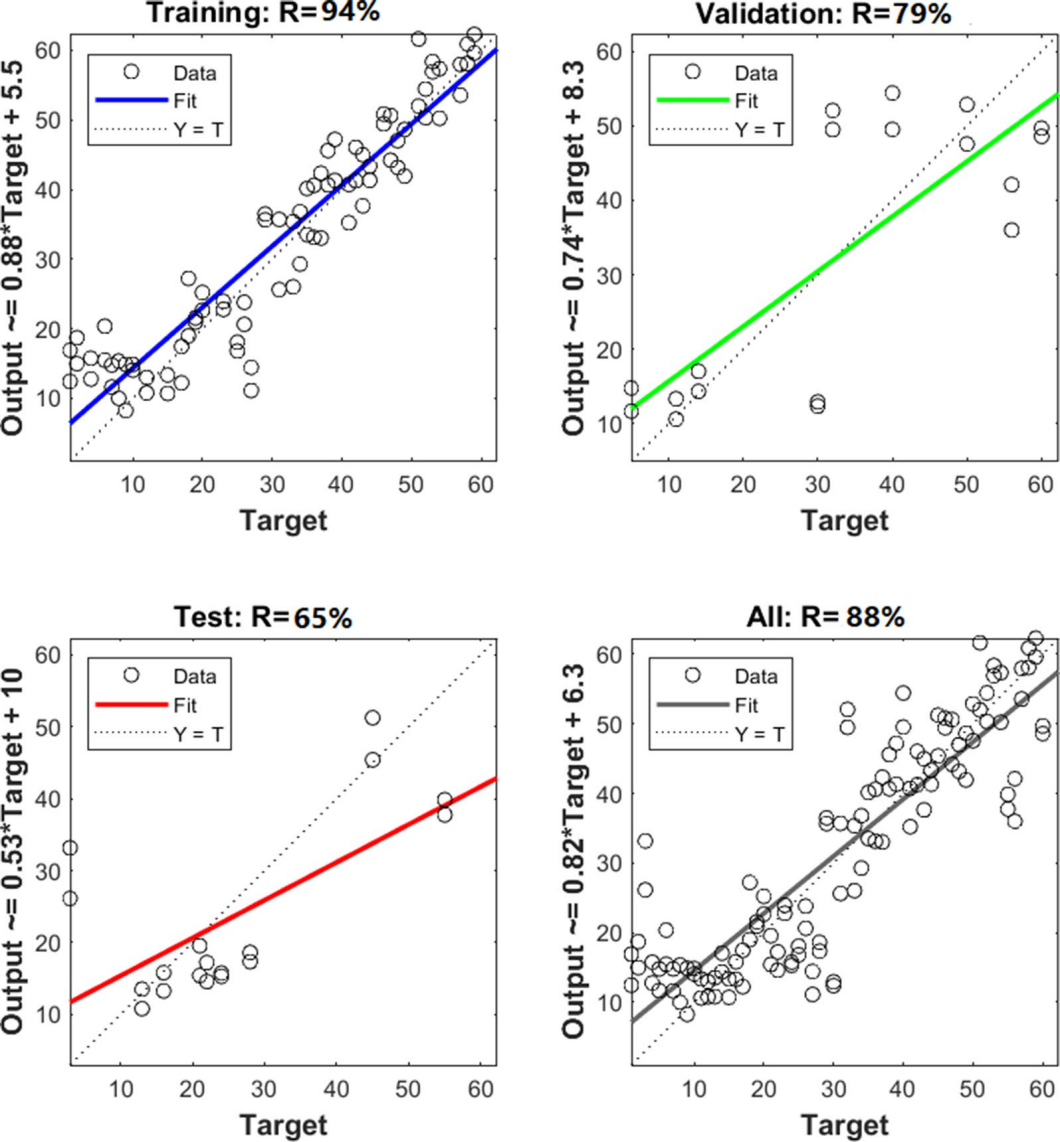
Regression plots during training, testing, and validation of ANN in Matlab showing the R values **a** without nanoparticles (LIBS) and **b** with nanoparticles (NELIBS)

The performance plot represents that the number of iterations increasing MSE becomes minimum. The plot also evaluates that validation and testing data set errors have almost similar characteristics, and the best performance of the model occurred at the selected number of iterations (epochs). The regression plot of the correlation coefficient (R) is presented in Fig. 10, showing the relationship between the outputs and targets for the ANN model.

## Discussion

Efforts are still developing to study microbial metabolism and physiology in the context of elemental composition. Major elements participate in the metabolic processes of microbial systems. Therefore, elements identified in this experiment by their specific spectral lines can’t all be related to the spectral fingerprint of a bacterial cell. Since C, H, N, O, P, and S elements are essential to forming the macromolecules of living cells, the proportion of such elements within the cell is conserved across all microorganisms. However, other elements are required to stabilize these molecules and maintain a habitable environment. In addition, certain inorganic elements and ions are relevant for the homeostasis of life. Therefore, some elements, such as Ca, Na, K, Cl, Mg, Fe, and Mn, are expected to fluctuate moderately, as they are relevant to bacterial homeostasis. Others, such as Zn, fluctuate significantly (Novoselov et al. 2013). Sodium, Potassium, Magnesium, and Calcium are considered the major elemental inorganic cations in a bacterial cell, as they are critical to many cellular functions. The abundance of these elements varies modestly among different bacterial cells. Magnesium is the most abundant element in many bacterial cells. It is essential in coordinating phosphoryl oxygen atoms, as a cofactor for multiple enzymes, and helps maintain the pH (Compton and Mindell 2010). Potassium plays a vital role in cell membrane transport (Doyle et al. 1998). Calcium is less abundant than magnesium; it serves as a cofactor for certain enzymes and a component of endospores (Burke and Slinker 1982). Also, it could coordinate with E.F. proteins and form a class of calcium-binding proteins that have essential physiological roles in some bacterial cells (Michiels et al. 2002). Sodium ions can participate in Na^+^/H^+^ antiporters preventing over-alkalinization of cytoplasm in stress conditions (Doyle et al. 1998).

Other elements like Fe^3+^ and Mn^4+^ act as final electron acceptors during respiration by some microorganisms^34^. Strontium can substitute calcium and magnesium in some processes like spore formation, polysaccharides, flagellum biosynthesis (Robinson et al. 1992), and enzyme activation (Goodwin et al. 1996). Zinc and transition metals like Vanadium, Manganese, Cobalt, Nickel, Copper, and Molybdenum comprise only 1–2% of the total microbial cell mass. Still, they are essential for cells functioning as catalytic centers for enzyme catalysts. Cobalt and Molybdenum present as trace elements are usually incorporated into vitamins and enzymes. For example, Cobalt is associated with vitamin B12 and Molybdenum in the bacterial nitrogenase enzyme. Chloride is a significant anion and a central halogen atom in many microbes that play a crucial role in osmoregulation and energy metabolism, especially for halophiles (Roeßler and Müller 2002).

It is noted that all bacteria spectra are mostly similar since all bacteria mostly contain the same elements, as seen in Fig. 3. However, the identification of bacterial samples and discrimination between them can be achieved based on the presence and absence of some elements’ spectral lines (Fig. 7). In addition, the relative signal intensity of detectable elements spectral lines in other bacteria could also be utilized for bacteria discrimination (Fig. 6). The matrix effect may affect the variations in the intensity of some detected elements’ spectral lines, where the presence of some elements alters the emission behavior of another (Cremers and Radziemski 2013; Noll 2012; Singh and Thakur 2007). The existence and absence of certain elements in different bacteria depend on several factors. Mainly the strain-specific metabolic processes, the interaction with the surrounding media on which the bacteria are grown, and various elemental contributions to the metabolic cycle of the organism. Lithium (Li^+^), for example, is toxic to numerous microbial organisms. However, in some cases, it substitutes Na^+^ in the co-transport of amino acids and some sugars (Chen et al. 1985) and drives the flagellar motor in some bacteria (Liu et al. 1990). Likewise, iodine can be incorporated into metabolites by bacteria. Even though iodine is well known to have essential roles in killing bacteria, many bacteria can accumulate iodine through different mechanisms (Yeager et al. 2017).

The potential improvement of Laser-induced breakdown sensitivity using silver nanoparticles is a huge step towards the rapid and accurate diagnostic detection and classification of bacteria in different settings with almost no sample preparation needed.

The reasons behind NELIBS enhancement have been explained in detail by Dell’Aglio et al. 2018, who showed the main differences between nano-enhanced LIBS and conventional LIBS are the different ablation and excitation processes that affect the characteristics of the laser-produced plasma. The field enhancement in LIBS produced by the nanoparticles deposited onto a semiconducting surface, silicon in the present case, is due to surface plasmon resonance (SPR). This may take place when the laser photons are in resonance with the local surface plasmon (LSP) or due to the effect of the high laser irradiance (> 1 GW./cm^2^) on the NPs. In the case of resonance with local surface plasmons, nanoparticle surface electron oscillation promotes the electromagnetic field leading to intense topical heating on the sample surface. On the other hand, in the case of a high laser irradiance, the nanoparticles break down, and the induced plasma energy is transferred to some of the samples in the vicinity of such nanoparticles (Abdel-Salam et al. 2018). However, the laser wavelength used in the present measurements (1064 nm) was not in resonance with the absorption peak (420 nm) of the used NPs (Abdel-Salam et al. 2018). Hence, the laser’s direct interaction with the NPs enhances LIBS intensity. Given the different plasma production mechanisms in LIBS and NELIBS, adopting other optimization procedures for the detection systems in the two techniques may be adequate. Nevertheless, there was not much difference between the detection optimized values (the delay time t_d_ and gate width ΔT) for LIBS and NELIBS measurements. Therefore, the spectra collected adopting both techniques in the present work were measured at the same values for t_d_ and ΔT. However, the factor of signal enhancement (ratio of NELIBS/LIBS for the intensity of the spectral lines) varied between both bacterial samples due to the matrix effect, i.e., the difference in the elemental composition proportions of each bacterial species.

In conclusion, the higher potential of NELIBS compared to the conventional LIBS in the discrimination between two bacterial strains with different taxonomic orders has been demonstrated. Furthermore, the results showed that biogenic AgNPs enhanced the spectroscopic sensitivity of NELIBS and facilitated the detection of various trace elements in both bacterial species. Furthermore, the spectral lines intensity enhancement factor, i.e. NELIBS/LIBS, revealed the effectiveness of using the NELIBS technique. Therefore, the discrimination between the two bacterial species has been achieved given the intensity difference of the spectral lines of some elements in both bacteria samples. Moreover, the presence of the spectral lines of certain elements in the spectrum of only one species of the two bacteria could also be used as a marker or a fingerprint characterizing such bacterial species. Moreover, the ANN model was created to evaluate the difference between the two bacterial species’ data sets, providing 88% and 92% differentiation accuracy for all data sets using LIBS and NELIBS, respectively. Compared to conventional microbiological discrimination techniques, this work demonstrated that different bacterial pathogens could be identified and classified at high precision using NELIBS with its pros. Namely, NELIBS is simple, needs no or minimum sample preparation, and is cost-effective.

## Supplementary Information

**Additional file 1: Figure S1.** TEM micrograph of the prepared silver nanoparticles.

## Data Availability

The data underlying the results presented in this paper are available from the corresponding author upon request.

## Abbreviations

LIBS: Laser-induced breakdown spectroscopy
NELIBS: Nanoparticle-enhanced laser-induced breakdown spectroscopy
ANN: Artificial neural networks
NPs: Nanoparticles
AgNPs: Silver nanoparticles
