# Supplementary figures and images for: Discriminating two bacteria via laser-induced breakdown spectroscopy and artificial neural network
Source: AMB Express. 2023 Jun 20;13:61. doi: 10.1186/s13568-023-01569-0 (PMC10281934; doi:10.1186/s13568-023-01569-0)

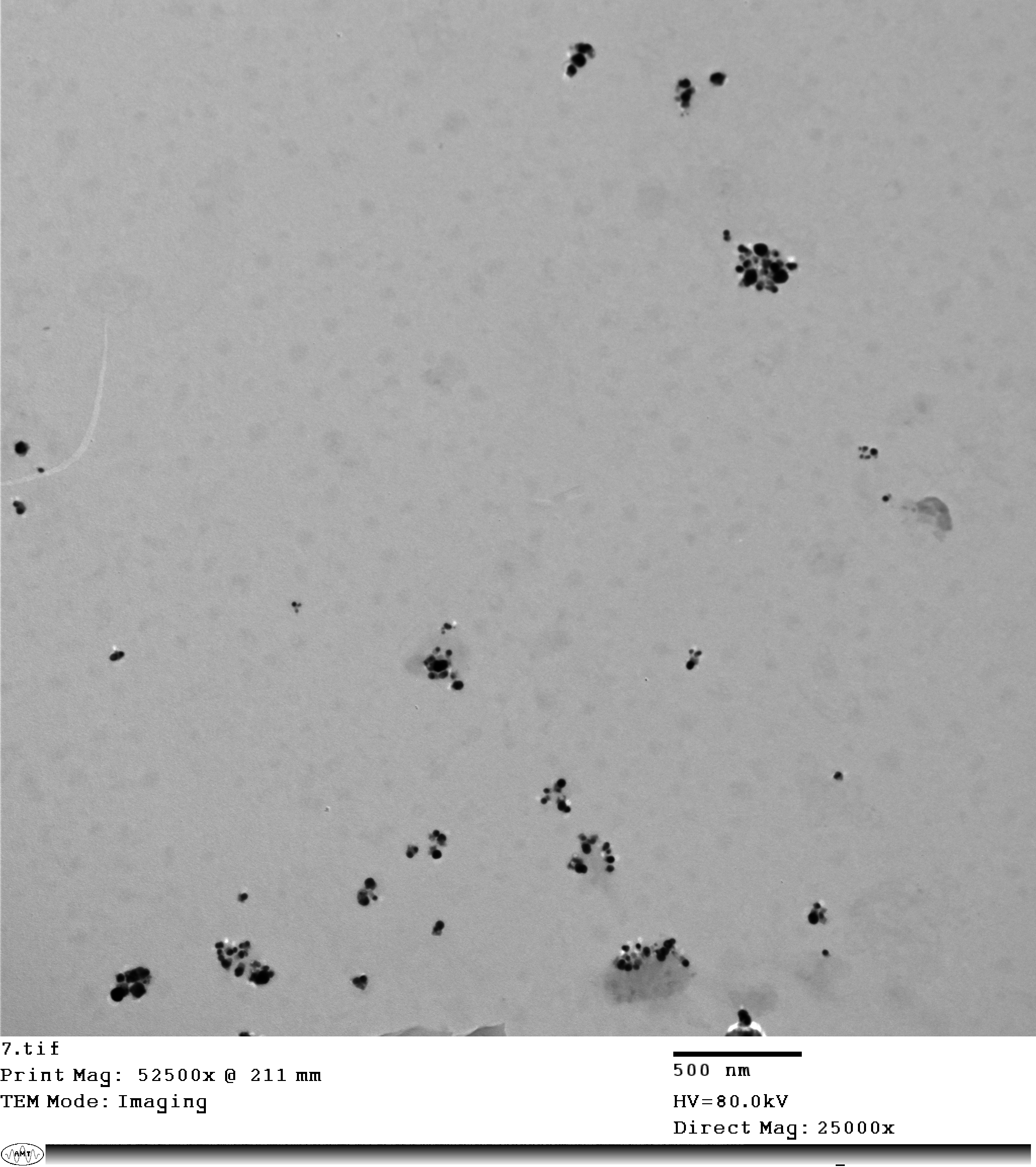

Supplement: Supplementary file 1 — Additional file 1: Figure S1. TEM micrograph of the prepared silver nanoparticles. [file 13568_2023_1569_MOESM1_ESM.tif]
